# Mixed-vs-Segregated Stack Polymorphism in the *N*,*N*,*N*′,*N*′-Tetramethylbenzidine-TCNQF_4_ Charge Transfer Complex

**DOI:** 10.1021/acs.jpcc.5c01376

**Published:** 2025-04-28

**Authors:** Elena Ferrari, Francesco Mezzadri, Matteo Masino

**Affiliations:** † Dipartimento di Scienze Chimiche, della Vita e della Sostenibilità Ambientale & INSTM-UdR Parma, Parco Area delle Scienze, 17/A, Parma 43124, Italy; ‡ Institute of Materials for Electronics and Magnetism-CNR, Parco Area delle Scienze, 37/A, Parma 43124, Italy

## Abstract

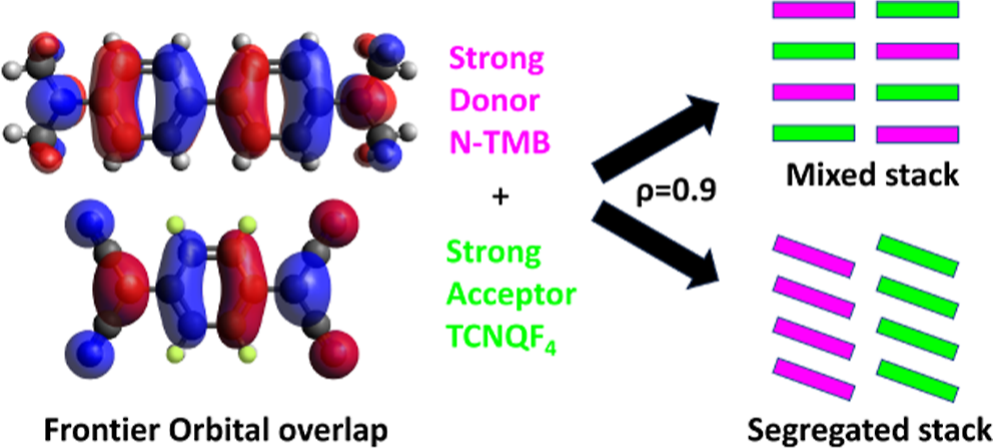

Organic charge transfer (CT) cocrystals are a wide class of 1-D materials based on stacks of planar π-electron donor (D) and acceptor (A) molecules overlapping their frontier orbitals due to the CT interaction. Most CT crystals have either a segregated or mixed stack motif: in segregated stacks, D and A molecules form separate columns, while in mixed stacks, D and A alternate along the same stack. Although CT crystals often display polymorphism, very few D–A pairs are known to crystallize with both stack motifs. Here, we present a new CT complex presenting both mixed and segregated stack phases, based on the strong donor *N*,*N*,*N*′,*N*′-Tetramethylbenzidine (N-TMB) and the strong acceptor TCNQF_4_. The combination of polarized IR and Raman spectroscopy with X-ray diffraction on single crystals found common structural and optical features between the two phases. The low temperature data also suggest that a stack distortion occurs in the segregated stack phase.

## Introduction

**Figure 1:**
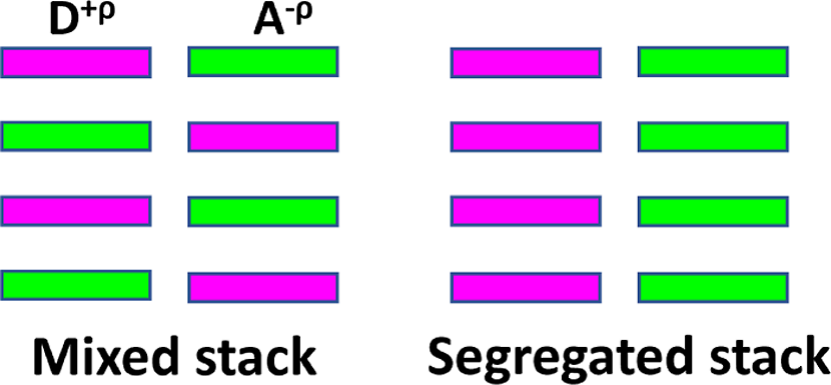
Schematic illustration of mixed and segregated stacking motifs of CT cocrystals.

Organic charge transfer (CT) crystals represent a unique class of low-dimensional functional materials based on planar π-electron donor (D) and acceptor (A) molecules. CT crystals exhibit a wide variety of electronic properties and structural patterns, with CT interactions playing a fundamental role in defining them. First, the peculiar directionality of the CT interactions, which dominates other intermolecular interactions, yields 1-D stacks. Structurally, the stack motifs fall into two broad categories: segregated and mixed stacks (MS), each with distinct transport and optical properties. In segregated stacks (SS), D and A form separate columns, whereas in MS, D and A molecules alternate along the same stack (Figure 1). Finally, the strength of CT interactions determines the ionicity ρ, which is the extent of electron transfer from D to A components, leading to neutral (ρ < 0.5) or ionic (ρ > 0.5) ground states.^1^ SS is always ionic, while the ground state of MS can be either neutral or ionic.

The stack motif strongly affects their optoelectronic properties. Indeed, SS crystals were first studied as organic metals after the discovery of TTF-TCNQ metallic conductivity in 1972.^2,3^ Subsequently, mixed-stack crystals attracted attention as ambipolar semiconductors and potential ferroelectrics and, more recently, for their tunable luminescence.^4,5^

The stacks are further classified into regular or dimerized. The intermolecular distances along the stack are equal in the regular, while two different distances alternate along the chain in the dimerized. Dimerization is always favored in ionic stacks due to Peierls instability. As a consequence, ionic regular stacks dimerize below a critical temperature, leading to the metal-to-insulator transition in SS organic conductors.^6^ On the other hand, neutral MS may undergo the neutral-to-ionic transition, a charge and structure instability where the ionicity increase is always associated with the stack dimerization.^7^ Such inversion symmetry breaking along the stack may also lead to electronic ferroelectricity.^8^

The electronic properties of CT crystals finally result from the interplay between the energy and symmetry of the D and A frontier orbitals and the 3-D molecular shape, controlling the intermolecular interactions and the crystal packing. Thus, the appropriate choice of CT pair allows for the rational design of new materials and the fine-tuning of their properties. In particular, the preferred stack motif depends mainly on the overlap effectiveness between D and A frontier orbitals.^9^ For these reasons, although polymorphism is quite common in CT crystals, very few D–A pairs are known to crystallize with both stack motifs.^10−13^

**Figure 2:**
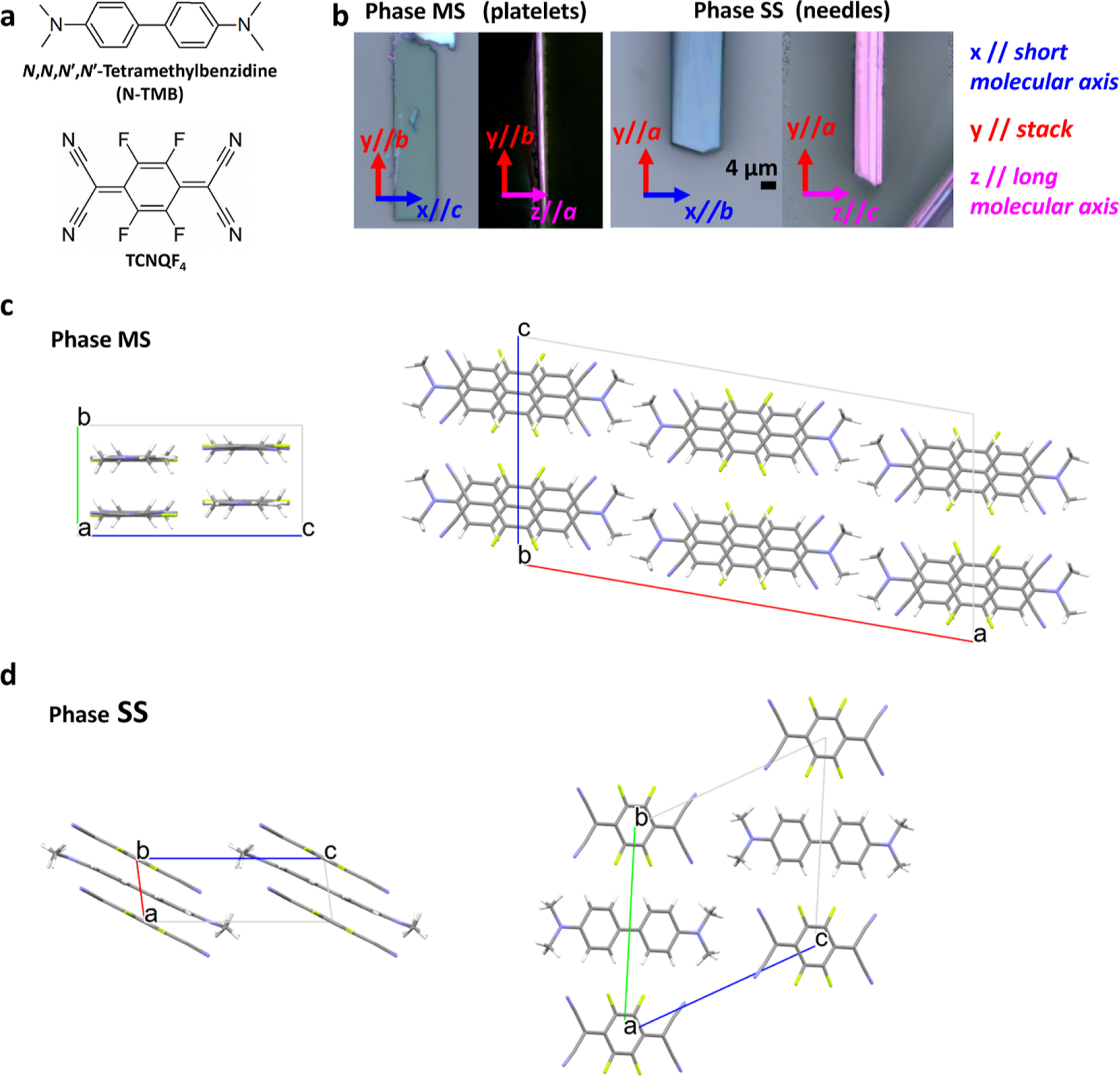
(a) Molecular structure of D and A. (b) Microscopic images of N-TMB-TCNQF_4_ crystals, laying on the blue *xy* and the purple *yz* planes and correlation between *x*,*y*,*z* and the molecular axes. (c) Phase MS unit cell, viewed along *a* (left) and *b* (right). (d) Phase SS unit cell, viewed along *b* (left) and *a* (right).

Here, we present a new D–A pair showing mixed-segregated stack polymorphism, based on the strong donor *N*,*N*,*N*′,*N*′- Tetramethylbenzidine (N-TMB) and the strong acceptor TCNQF_4_ (Figure 2a). The two N-TMB-TCNQF_4_ polymorphs were investigated using polarized IR and Raman spectroscopy on oriented single crystals as a structural tool combined with X-ray diffraction. While Phase MS has a mixed dimerized stack with a monoclinic unit cell, Phase SS is a segregated regular stack with a triclinic unit cell.

## Methods

### Crystal Growth

Phase MS single crystals were obtained by physical vapor transport (*PVT*). The two pure components were placed in an open quartz tube under nitrogen flow, following the lines of ref (14). After 1 day, many thin, blue, elongated, rectangular platelets (typical size 1 × 0.5 × 0.002 mm^3^) were found in the colder zone of the tube. If observed on the lateral face, the crystals display a bright purple color (Figure 2b, left).

Phase SS crystals were grown by slowly cooling hot acetonitrile solutions of the two components in a 1:1 stoichiometric ratio. Due to the strong tendency to precipitate, the starting solutions (60 μL, *c* = 10^−2^M) were added to 10 mL of solvent at 75 °C. The vial was then placed in a Dewar filled with hot water and left to cool to room temperature. Very small needle-like crystals (typical dimensions 0.5 × 0.02 × 0.01 mm^3^) grew in a day. The crystals display extended faces of two different colors, bright purple and blue, as in Phase MS, and are always twinned along the elongation direction (Figure 2b, right).

### Spectroscopic Characterization

The IR spectra were recorded with a Bruker IFS-66 FT-IR spectrometer coupled to a Hyperion 1000 IR microscope. The thinnest crystals were selected for the absorbance measurements and then oriented on a ZnSe slide, which was also used as a reference. The radiation was polarized parallel or perpendicular to the stack direction, identified due to the presence of the CT excitation above 4000 cm^−1^. In the case of Phase SS needles, the spectra were recorded on both developed faces, while for Phase MS platelets, only the extended blue face was accessible.

The Raman spectra were measured in backscattering geometry with a Horiba LabRAM HR Evolution spectrometer equipped with a 633 nm HeNe laser and a ULF Bragg filter to reject the Rayleigh scattered radiation (lowest accessible frequency 4 cm^−1^). The laser power was always set below 0.1 mW to avoid sample heating. In both phases, the polarized Raman spectra were collected on either the blue or the purple face containing the stack axis, with the exciting and scattered light polarized parallel or perpendicular to the stack. The thin purple face of the Phase SS platelets was assessed by fixing an oriented crystal between two thin glass slides.

The samples were cooled to 80 K using a Linkam HFS 91 stage fitted under the IR and Raman microscopes.

### Crystal Structure Determination

Single-crystal X-ray diffraction experiments were carried out using a Bruker D8 Venture instrument equipped with a Photon II 2D detector and a Cu microsource. Data were collected at 200 K in order to reduce the thermal parameters after the presence of phase transitions in the 300–200 K range was ruled out both by X-ray diffraction and Raman spectroscopy. Crystal structure solution and refinement were carried out by using the SHELXT^15^ and SHELXL^16^ programs, respectively.

### Calculations

Standard DFT computational methods (B3LYP, 6–31G(d)) were exploited for the calculation of equilibrium geometry and vibrational frequencies of N-TMB, in both the neutral and ionized state, using Gaussian 16 B.10.^17^ The frequencies were scaled by the factor 0.9613, as suggested in ref (18).

## Results and Discussion

### Crystal Growth and Structure

CT complexes containing N-TMB and TCNQ derivatives have remained elusive, likely due to the difficult crystal growth. First, the solution growth is limited by the poor solubility and the resulting tendency to precipitate. Then, *PVT* requires different sublimation temperatures for N-TMB and TCNQF_4_. Finally, this CT pair forms two polymorphic phases depending on the growth conditions. Phase SS could be selectively obtained from solution by using polar organic solvents. Differently, nonpolar solvents such as toluene gave a flaky Phase MS precipitate. *PVT* growth yielded only Phase MS as well with improved crystal quality. A possible explanation for the selectivity of polar solvents toward Phase SS is the stabilization of radical ion self-dimers in solution and their subsequent crystallization.^19^

The unit cell of Phase MS can be indexed in the monoclinic crystal system and lattice parameters *a* = 27.122(3); *b* = 6.4911(7), *c* = 13.3777(13) Å, and β = 99.756(7)°, while the extinctions observed (*h* + *k* + *l* with *h* + *k* = 2*n* and *h*0*l* with *h*, *l* = 2*n*) indicate the *C*2/*c* space group. Structure solution reveals a dimerized stack (Figure 2c and Table 1) with 4 D–A pairs in the unit cell. The molecular planes lie approximately within the (010) plane, with the short in-plane molecular axes both aligned to *c*. The A and D moieties alternate along the [010] stacking direction at distances of 3.172 and 3.319 Å (plane-centroid). Such a face-to-face arrangement favors the overlap between the molecular orbitals involved in the CT (Figures S7 and S8) and was observed in all the known mixed stack CT crystals containing TCNQ and benzidine derivatives.^20−24^

**Table 1:** Structural Parameters of the Two Polymorph Phases at 200 K

	phase MS	phase SS
space group	*C*2/*c*	*P*−1
*a* (Å)	27.122(3)	3.9086(4)
*b* (Å)	6.4911(7)	12.7716(13)
*c* (Å)	13.3777(13)	12.9571(13)
α(deg)	90	62.399(6)
β(deg)	99.756(7)	83.168(7)
γ(deg)	90	89.358(7)
V (Å^3^)	2321.1(4)	568.418
Z	4	1
R1(F_*o*_ > 4σ(F_*o*_))	9.50	17.44

Face indexing of a platelet found the extended crystal face parallel to the *bc* plane, suggesting a growth driven by strong and directional intermolecular interactions along *b* and *c*. Indeed, the strongest intermolecular forces are the CT interaction along the *b* stack axis and the F–H hydrogen bonds between A and D molecules of adjacent stacks, acting along *c*. The interactions are weaker along *a* due to the bulky nonpolar N(CH_3_)_2_ groups.

The crystal structure determination for Phase SS implied relevant difficulties related to the low quality of the crystals, always characterized by the presence of twinning and severe mosaicity. Both the coexistence of randomly oriented crystallites and twins obeying the law (1 0 0; 0 −1 0; 0.73 0 −1) are detected, regardless of the synthesis technique and parameters exploited, finally resulting in strongly reduced crystallite size and low diffraction intensity, in particular at high sin θ/λ. As a result, the poor statistics of the high-resolution data do not allow a reliable refinement of the fine details of the structure. On the other hand, the quality of the low-resolution data is suitable for the determination of lattice parameters, space group symmetry, and molecular arrangement.

Indeed, the obtained 3D diffraction pattern can be indexed with a triclinic lattice having *a* = 3.9086(4); *b* = 12.7716(13); *c* = 12.9571(13) Å; α = 62.399(6); β = 83.168(7); and γ = 89.358(7)°. Structure solution gives the best results in the centrosymmetric *P* –1 space group, unambiguously indicating the unit cell to contain one D and A couple (Figure 2d and Table 1). It should be noted that, given the poor data quality, a slight departure from the centric symmetry, if present, could not be detected; however, no doubts arise regarding the molecular stacking. Indeed, differently from Phase MS, the D and A molecules separate here into SS running parallel to the *a* axis. The intermolecular distances along the D and A stacks are, respectively, 3.35 and 3.20 Å, as the molecular planes are tilted by 31 and 35° with respect to the stack direction. Such ring-overbond overlap is common in CT complexes containing TCNQ and TCNQF_4_.^11,25,26^ In the case of N-TMB stacks, the tilt also reduces the steric interactions between the N(CH_3_)_2_ groups of adjacent molecules along the stack (Figure S8). Despite the different stack motifs, the two phases share common structural features, driven by the interplay between frontier orbital symmetry, elongated molecular shape, steric interactions, and directional F–H hydrogen bonds. First, in both phases the overlaps along the stack are ring-overbond type. Then, the short and long in-plane molecular axes are respectively aligned.

### Ionicity Determination

**Figure 3:**
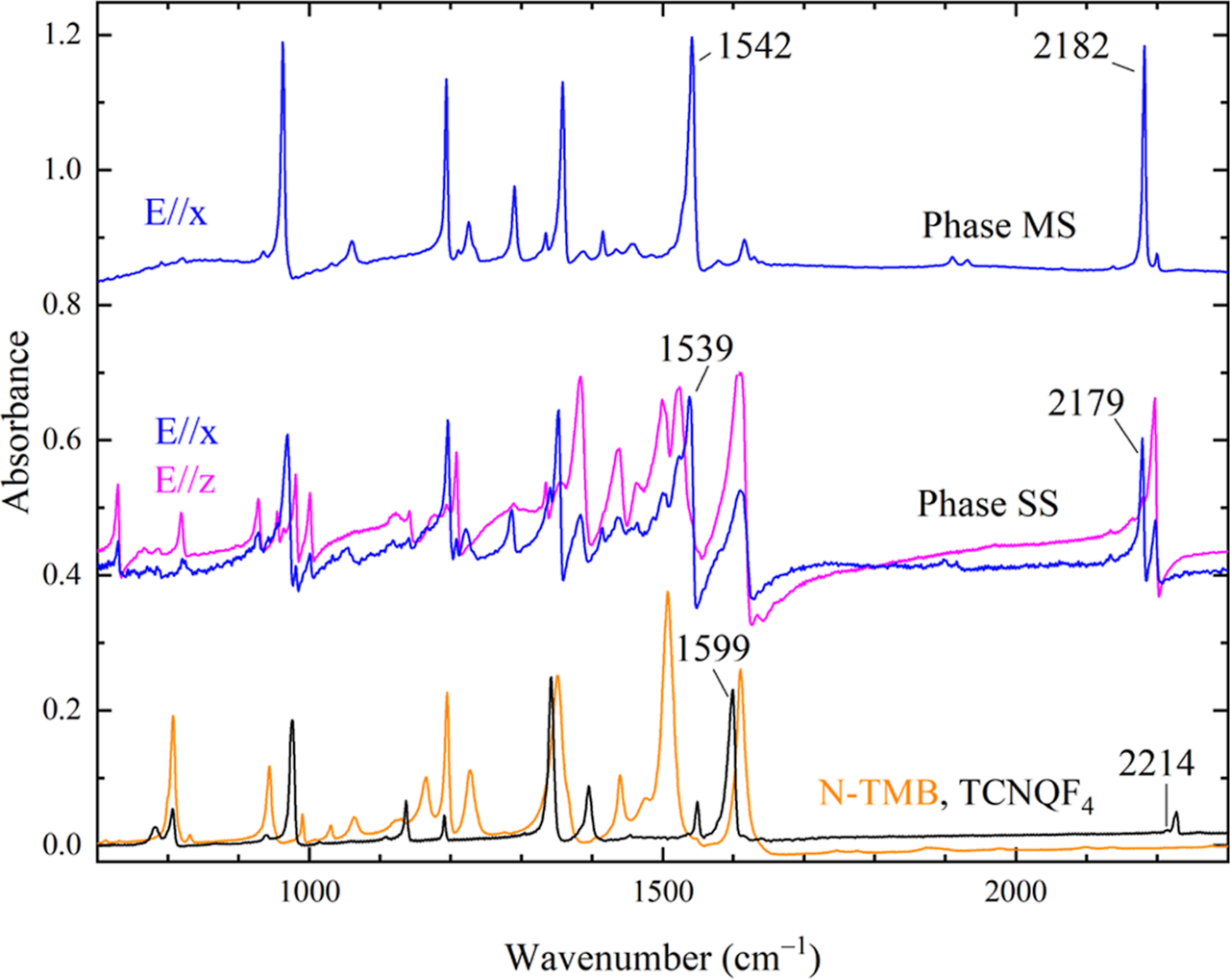
IR spectra of the two N-TMB-TCNQF_4_ phases, polarized perpendicular to the stack direction *y*. The powder spectra of the reagents are shown for comparison, and the spectra of each phase are offset for clarity.

Consistent with the structural similarity, the crystals of the two phases share many optical features. First, a CT band is present in the near infrared (5000–7000 cm^−1^), completely polarized along the stack direction (Figure S1). Then, the crystals of both phases display a blue face and a bright purple face. Finally, the polarized IR and Raman spectra of the two phases measured on crystal faces of the same color show very similar patterns (Figures 3 and 6).

Indeed, in both phases, the blue face contains the short molecular axes, while the purple face is almost parallel to the long ones. For this reason, the spectra of the two phases can be conveniently analyzed together in a common reference system *xyz*, where *y* coincides with the stack direction and *x* and *z* are perpendicular to it and are close to the short and long molecular axes, respectively (Figure 2b). These directions correspond, respectively, to *b*, *c*, and *a* in Phase MS and to *a*, *b*, and *c* in the case of Phase SS.

In the case of the thin phase MS platelets, IR measurements were possible on the extended *xy* (*bc*) plane only. As expected, the *x*-polarized spectrum, with polarization along the short molecular axes, is dominated by the TCNQF_4_*b*_2*u*_ modes (Figure 3 and Table 2). Indeed, in the *D*_2*h*_ point group of the TCNQF_4_ molecule, the *b*_1*u*_ and *b*_2*u*_ modes have the same symmetry of the long and the short in-plane axes, respectively. The charge-sensitive ring C=C stretching mode *b*_2*u*_ν_33_, occurring at 1542 cm^−1^, is red-shifted by 57 cm^−1^ with respect to the neutral TCNQF_4_ molecule, corresponding to ρ = 0.92.^27,28^ Also, the ionicity estimated by the two C≡N stretching bands *b*_2*u*_ν_32_ and *b*_1*u*_ν_19_ is consistent with a system close to full ionicity.

**Table 2:** Band Assignment of the Perpendicular Polarized IR Spectra of the Two Phases at Room Temperature^a^

assignment	phase MS	phase SS	phase SS
	*E*//*x*	*E*//*x*	*E*//*z*
TCNQF_4_, *b*_2*u*_ν_36_	962	967	
TCNQF_4_, *b*_1*u*_ν_21_			1143
TCNQF_4_, *b*_2*u*_ν_35_	1194	1196	
N-TMB, δ CH (*z*)			1208
N-TMB, δ CH (*x*)	1289	1287	
TCNQF_4_, *b*_2*u*_ν_34_	1359	1352	
N-TMB, ν N-ring (*z*)			1384
N-TMB, δ CH_3_			1438
TCNQF_4_, *b*_1*u*_ν_19_			1499
N-TMB, δ CH + ν ring (*z*)			1522
TCNQF_4_, *b*_2*u*_ν_33_	1542	1539	
N-TMB, ν ring + δ CH (*z*)		1607	1607
TCNQF_4_, *b*_2*u*_ν_32_	2182	2179	
TCNQF_4_, *b*_1*u*_ν_18_		2197	2197

a The assignments are based on refs (27 and 28) for TCNQF_4_ and on the DFT calculation for N-TMB (Figure S9). In the case of N-TMB assignments, *x* and *z* indicate short and long axis polarized vibrations, respectively. All the frequencies are in cm^−1^.

In Phase SS, the IR spectra polarized perpendicular to the stack measured on the two faces are different. The spectrum measured with the light polarized along *x* is very similar to the corresponding spectrum of Phase MS, with very strong TCNQF_4_*b*_2*u*_ bands. Differently, the *z*-polarized spectrum displays the TCNQF_4_*b*_1*u*_ modes, together with the main N-TMB bands. The charge-sensitive TCNQF_4_*b*_2*u*_ν_33_ peaks at 1539 cm^−1^, indicating ρ = 0.95 and the two C≡N stretching bands *b*_2*u*_ν_32_ and *b*_1*u*_ν_19_ again confirm this result. Thus, N-TMB-TCNQF_4_ Phase SS is also ionic. Such a high ρ value is due to the strong donor and acceptor character of the two molecules.^29,30^

As in the case of the polarized IR spectra of the two phases, the Raman spectra follow the same pattern (Figure S2). The totally symmetric intramolecular vibrations are amplified by an order of magnitude with the *zz* polarization. This is a clear resonance effect since the 633 nm exciting line is close to the long molecular axis-polarized absorptions of both radical ions (Figure S3).^31,32^ Differently, the *yy* polarized spectra are stronger at low frequencies.

Among the few known 1:1 CT crystals presenting MS-SS polymorphism, this is the first case of a similar ionicity in the two phases. In all these CT systems, the SS phases are ionic, while the MS ones are neutral, due to the inefficient overlap between the frontier orbitals of the two molecules.^10−13,33−35^ On the contrary, both phases are ionic in this case. Indeed, the N-TMB HOMO and the TCNQF_4_ LUMO have the same symmetry with respect to the inversion centers of the corresponding molecules (Figure S7), resulting in an effective overlap also in the MS phase.

### Stack Motif and Symmetry

While the IR spectra polarized perpendicular to the stack give information about the degree of CT, the parallel polarized one is very sensitive to the stack motif and symmetry. Both features have typical spectroscopic signatures due to the coupling between the totally symmetric intramolecular vibrations with the CT electronic excitations polarized along the stack direction (electron-molecular vibration or e-mv coupling). When the molecular sites lose the inversion symmetry as a consequence of stack dimerization, the Raman-active, totally symmetric modes also become IR-active, with strong intensity and polarization along the stack. Another effect of the e-mv coupling is the frequency decrease of the coupled modes. Differently, regular stacks do not display IR vibronic features.^36−38^

The comparison between IR and Raman spectra also allows us to distinguish dimerized MS from SS (Figure S4). In dimerized MS, the totally symmetric modes are both IR and Raman active with the same frequency, lowered by the e-mv interaction. Differently, in dimerized SS, the IR vibronic bands appear at a frequency lowered with respect to their unperturbed Raman counterparts. This is due to the inversion center located between identical ions, defining a symmetric and an antisymmetric combination of the totally symmetric molecular modes. Only the IR-active antisymmetric one is coupled with the CT excitation, while the Raman-active symmetric counterpart is not.^37,39^

**Figure 4:**
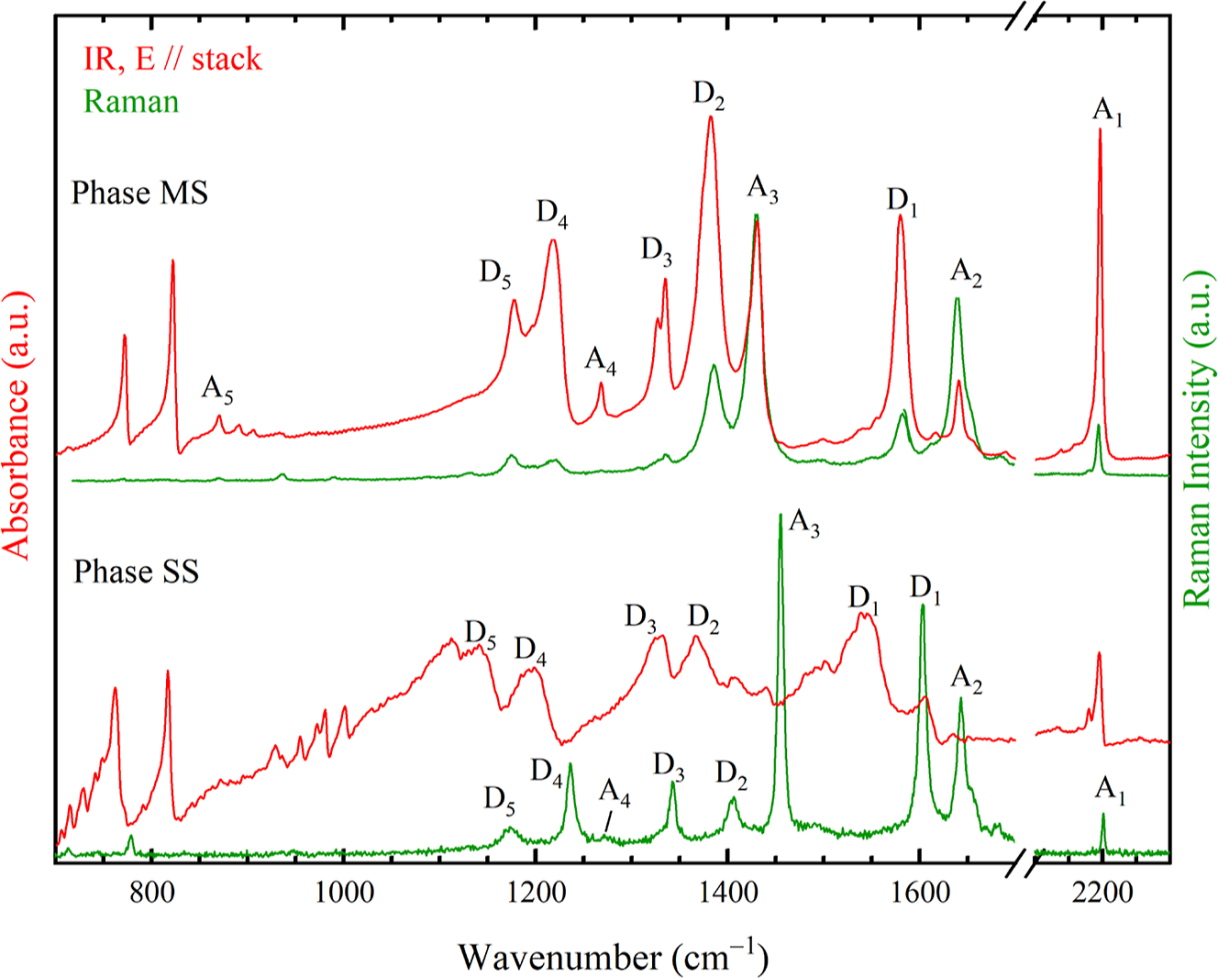
Comparison between Raman and parallel polarized IR spectra of the two phases. The letters correspond to the assignments in Table 3 and D and A, respectively, indicate N-TMB and TCNQF_4_ modes. The two strong IR bands around 800 cm^−1^ are due to out-of-plane C–H and C–F bending modes of N-TMB and TCNQF_4_. The phase MS spectra are upshifted for clarity.

The phase MS IR spectrum polarized along the stack displays many strong bands (Figure 4, upper panel, and Table 3) at the same frequencies of the totally symmetric N-TMB and TCNQF_4_ bands, visible in the Raman spectrum. All the totally symmetric Raman bands are red-shifted in Phase MS with respect to the corresponding unperturbed modes in Phase SS. The effect is stronger for the most coupled bands, as TCNQF_4_*a*_g_ν_3_, observed at 1431 and 1456 cm^−1^ in Phases MS and SS.^27^ These facts completely agree with the dimerized MS structure found by XRD.

**Table 3:** Frequencies of the Totally Symmetric Modes in the Raman and Parallel Polarized IR Spectra of the Two Polymorphs at Room Temperature^a^

assignment	phase MS	phase SS	phase SS
	IR and Raman	IR	Raman
A_1_: TCNQF_4_, *a*_*g*_ν_1_	2195		2202
A_2_: TCNQF_4_, *a*_*g*_ν_2_	1641		1644
D_1_: N-TMB, ν ring + δ CH (sym)	1580	1545	1604
A_3_: TCNQF_4_, *a*_*g*_ν_3_	1431		1456
D_2_: N-TMB, ν N-ring (sym)	1383	1367	1405
D_3_: N-TMB, ν ring–ring	1335	1330	1344
A_4_: TCNQF_4_, *a*_*g*_ν_4_	1271		1273
D_4_: N-TMB, δ CH (sym)	1267	1195	1237
D_5_: N-TMB, ρ CH_3_	1177	1141	1174
A_5_: TCNQF_4_, *a*_*g*_ν_5_	870		

a The assignments are based on refs (27 and 32) for TCNQF_4_ and N-TMB, respectively. All the frequencies are in cm^−1^.

Also, the parallel polarized mid-IR spectrum of Phase SS displays vibronic absorptions (Figure 4, bottom panel, and Table 3). However, in contrast to Phase MS, these bands are assigned to the N-TMB^+^ totally symmetric modes only, and their frequencies are lowered with respect to the corresponding Raman frequencies.^32^ This is consistent with the SS structure but suggests dimerization or, more likely, disorder along the N-TMB stacks. Although the N-TMB stack dimerization would involve a cell doubling along the stack, vibrational spectroscopy is more sensitive to the local molecular symmetry than XRD. Indeed, some CT crystals present the same discrepancy between IR vibronic features and a regular stack XRD structure.^40,41^ The absence of the TCNQF_4_^-^ totally symmetric modes in the Phase SS IR spectra indicates that the TCNQF_4_^-^ stacks are regular instead.

Finally, in Phase SS, the N-TMB and TCNQF_4_ Raman bands peak at nearly the same frequencies as the corresponding radical ions.^27,32^ This is consistent with the IR ionicity estimate, as in SS, the Raman frequencies are unperturbed by e-mv coupling.

### Lattice Phonons

The main differences between the spectra of the two polymorphs are found in the lattice phonon pattern, visible in the THz Raman spectra. The lattice vibrations, dependent on the intermolecular interactions of a specific crystal packing, are a fingerprint of the crystal phase. Furthermore, the number of phonon bands and their polarization reflect the unit cell multiplicity and symmetry.

**Figure 5:**
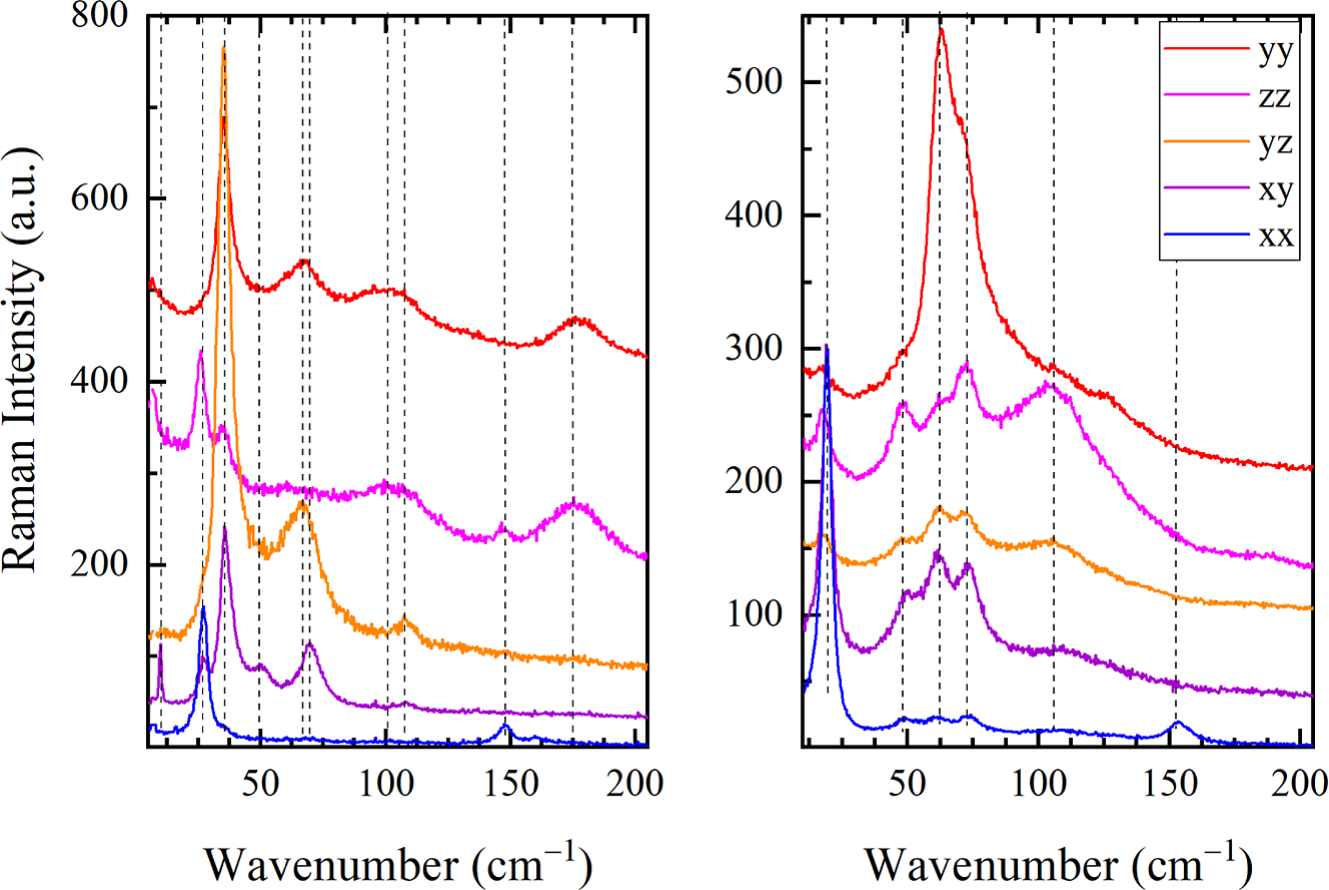
Polarized low-frequency Raman spectra of N-TMB-TCNQF_4_, Phase MS (left panel) and Phase SS (right panel). The spectra are offset for clarity.

Overall, the phase MS spectra display 11 bands below 200 cm^−1^, four of which are visible only with crossed polarizations (Figure 5, left panel). This is consistent with the predictions based on the *C*2/*c* unit cell factor group analysis: due to the lattice centering 12 phonon bands are expected as the primitive cell contains *Z* = 2 pairs, four of them belong to *A*_g_ symmetry and eight to *B*_g_ symmetry and are visible only in crossed polarizations (*xy* and *yz*). In the case of Phase SS, six phonons are present instead, active with both parallel and crossed polarizations (Figure 5, right panel), in agreement with the triclinic unit cell containing *Z* = 1 D–A pairs.

However, there are common features between the spectra of the two phases. In both phases, the *zz* polarized spectra are characterized by broad and overlapped bands, covering the whole 5–200 cm^−1^ range, that indicate structural disorder along the *z* direction. The most likely explanation is that the N-TMB axial N-(CH_3_)_2_ groups cause disorder along the *z* direction, selectively. On the contrary, the *xx* polarized spectra display very narrow bands. Thus, the intermolecular interactions have similar strength and directionality in the two N-TMB-TCNQF_4_ polymorphs, despite their different stack motifs.

### Temperature Behavior

The thermal behavior of both phases was investigated from 80 K to the decomposition temperature, 440 K in Phase MS and 390 K in Phase SS. Interconversion between the two polymorphs was never observed. Indeed, the transformation from an SS to an MS structure would involve a very high activation barrier.

In both polymorphs, the low-temperature evolution of the lattice phonons is continuous and involves strong line shape narrowing and little frequency hardening, consistent with a reduced thermal disorder. In Phase MS, the number of phonon bands is unchanged, indicating that the lattice is almost temperature independent (Figure S5). Also, the IR spectra do not show appreciable changes (Figure S6). The complete assignment of the totally symmetric bands of N-TMB and TCNQF_4_ in the two phases at 80 K is shown in Table S1.

**Figure 6:**
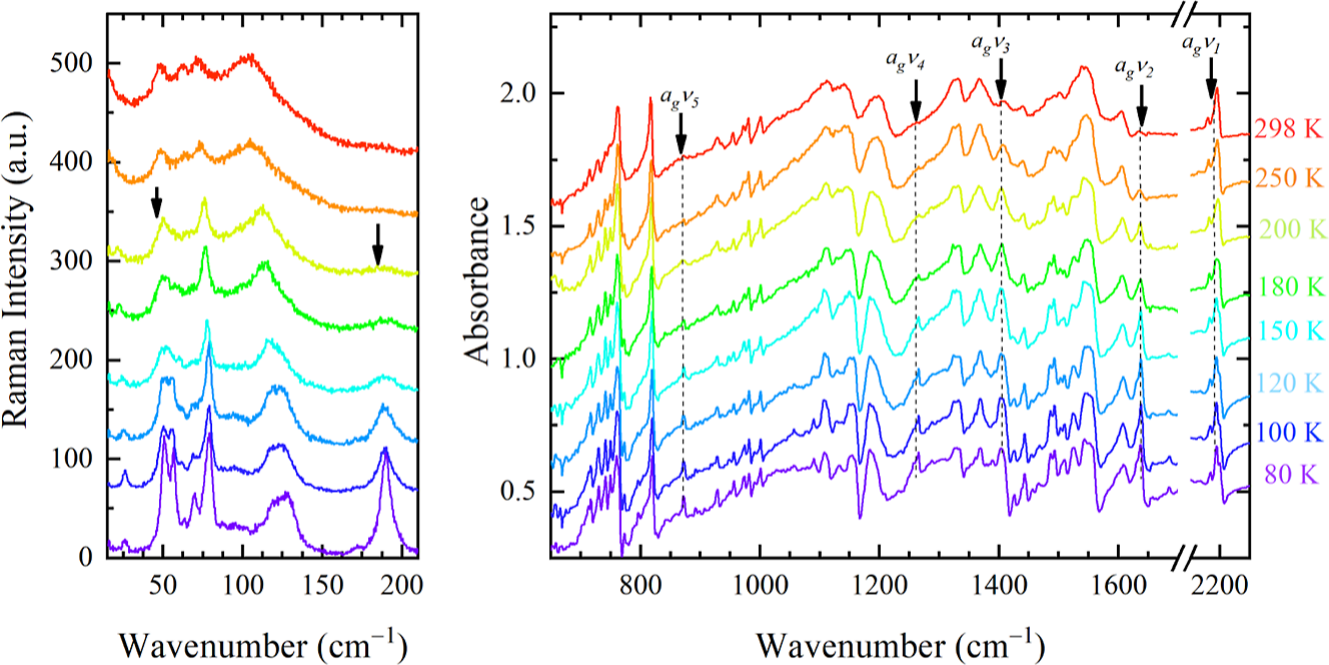
Left panel: low temperature Raman spectra of N-TMB-TCNQF_4_ Phase SS in the lattice phonon range. The exciting and scattered light are both polarized along *z*. The spectra are offset for clarity. Right Panel: temperature-dependent IR spectra polarized along the stack. In both graphs, the arrows mark the new bands.

On the contrary, in Phase SS, new bands appear around 200 K and gradually gain intensity on cooling, suggesting some kind of symmetry breaking (Figure 6, left panel). Overall, 12 bands can be resolved in all the polarizations, a number of phonons which is not consistent with the *Z* = 1 unit cell.

Also, the Phase SS IR spectra polarized along the stack display new vibronic absorptions at 2192, 1638, 1408, 1263, and 872 cm^−1^, appearing around 250 K, amplified on cooling (Figure 6, right panel, and Table S1). These bands are easily assigned to the TCNQF_4_ totally symmetric modes.^27^ Their IR activation demonstrates the loss of inversion symmetry of the TCNQF_4_ sites. Thus, at room temperature, the molecular sites locally lack inversion symmetry in the N-TMB stacks only, probably due to disorder. At low temperature, this also occurs along the TCNQF_4_ stacks, suggesting a long-range stack dimerization, consistent with the increased number of phonon bands. Indeed, in a regular segregated stack, the dimerization would involve the doubling of the unit cell along the stack direction.

## Conclusions

We grew and characterized two novel polymorph phases of the *N*,*N*,*N*′,*N*′-Tetramethylbenzidine-TCNQF_4_ CT compound, with unique structural features. The two polymorphs share similar ionicity and relative molecular orientations but have different stack motifs: mixed in Phase MS and segregated in Phase SS. Phase MS has a dimerized stack at all temperatures, while Phase SS likely undergoes dimerization below 200 K.

The low accuracy of the structural data was overcome by a detailed analysis of the polarized vibrational spectra, providing the main crystal packing features and the degree of CT ρ. IR and Raman have thus proven to be powerful structural tools to characterize molecular materials, which is fundamental when the available crystals are very small and/or twinned, preventing a complete X-ray analysis.

The unique N-TMB-TCNQF_4_ polymorphism was ascribed to the high electron donor and acceptor strengths of the component molecules and their efficient frontier orbital overlap. According to the previous studies, analogue ionic systems like TMPD-TCNQ,^41^ TMB-TCNQF_4_,^21^ and TMPD-TCNQF_4_^39,42^ crystallize with either MS or SS motif. Thus, these systems might also present different polymorph phases depending on the growth conditions. Further studies are needed to understand the nucleation and crystal growth mechanism of this and analogous CT pairs in different environments. This would also lead to an improvement in both the crystal quality and the electronic and optical properties of the resulting materials.

## Supporting Information Available

Supplementary IR and Raman spectra; DFT-calculated eigenvectors of selected vibrational modes of the N-TMB neutral molecule and its radical cation in the gas phase; and shape and symmetry of the frontier orbitals of the two molecules (PDF)
UTF-8N-TMB_TCNQF4_PhaseMS_200K (CIF)
UTF-8N-TMB_TCNQF4_PhaseSS_200K (CIF)

## Supplementary Material

jp5c01376_si_001.pdf

jp5c01376_si_002.cif

jp5c01376_si_003.cif
