# Supplementary material for: Mixed-vs-Segregated Stack Polymorphism in the N,N,N′,N′-Tetramethylbenzidine-TCNQF4 Charge Transfer Complex
Source: J Phys Chem C Nanomater Interfaces. 2025 Apr 28;129(18):8654–62. doi: 10.1021/acs.jpcc.5c01376 (PMC12067598; doi:10.1021/acs.jpcc.5c01376)
Supplement: Supplementary file 1 — jp5c01376_si_001.pdf [file jp5c01376_si_001.pdf]

Supporting Information for "Mixed-vs-Segregated stack polymorphism in N,N,N',N'-Tetramethylbenzidine-TCNQF$_4$ charge transfer complex"

Elena Ferrari,*,†,‡ Francesco Mezzadri,† and Matteo Masino*,†

†*Dipartimento di Scienze Chimiche, della Vita e della Sostenibilità Ambientale & INSTM-UdR Parma, Parco Area delle Scienze, 17/A, 43124, Parma, Italy*

‡*IMEM-CNR, Parco Area delle Scienze, 37/A 43124 Parma, Italy*

E-mail: elena.ferrari@imem.cnr.it; matteo.masino@unipr.it

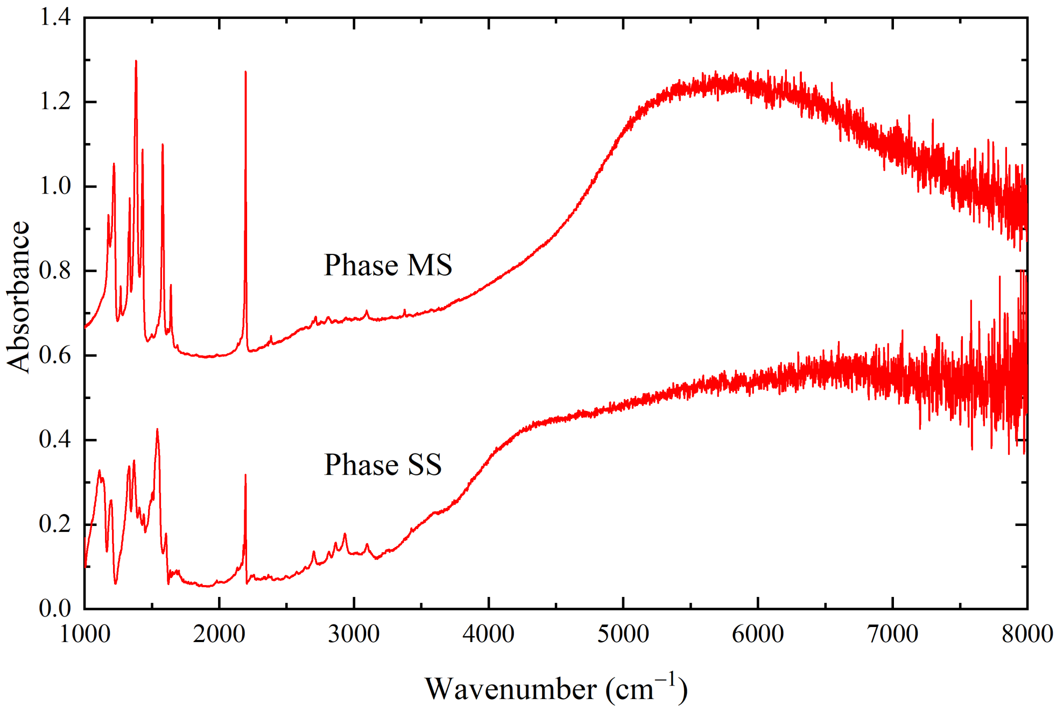

Figure S1: *Extended IR spectra of the two N-TMB-TCNQF*$_4$ *phases polarized along the stack. In the case of Phase SS the CT band saturates the spectrum due to the crystal thickness. The Phase MS spectrum is upshifted for clarity.*

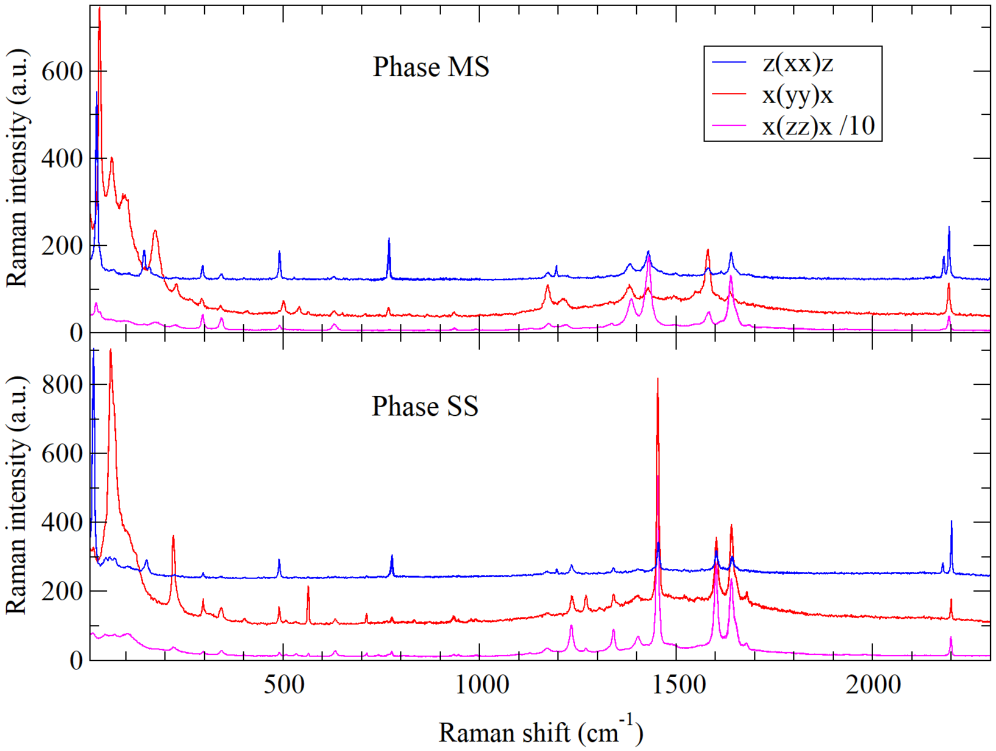

Figure S2: *Comparison between the polarized Raman spectra of the two N-TMB-TCNQF*$_4$ *crystal phases. For the sake of clarity, the three polarizations are offset and both the zz ones, enhanced by resonance, have been divided by 10.*

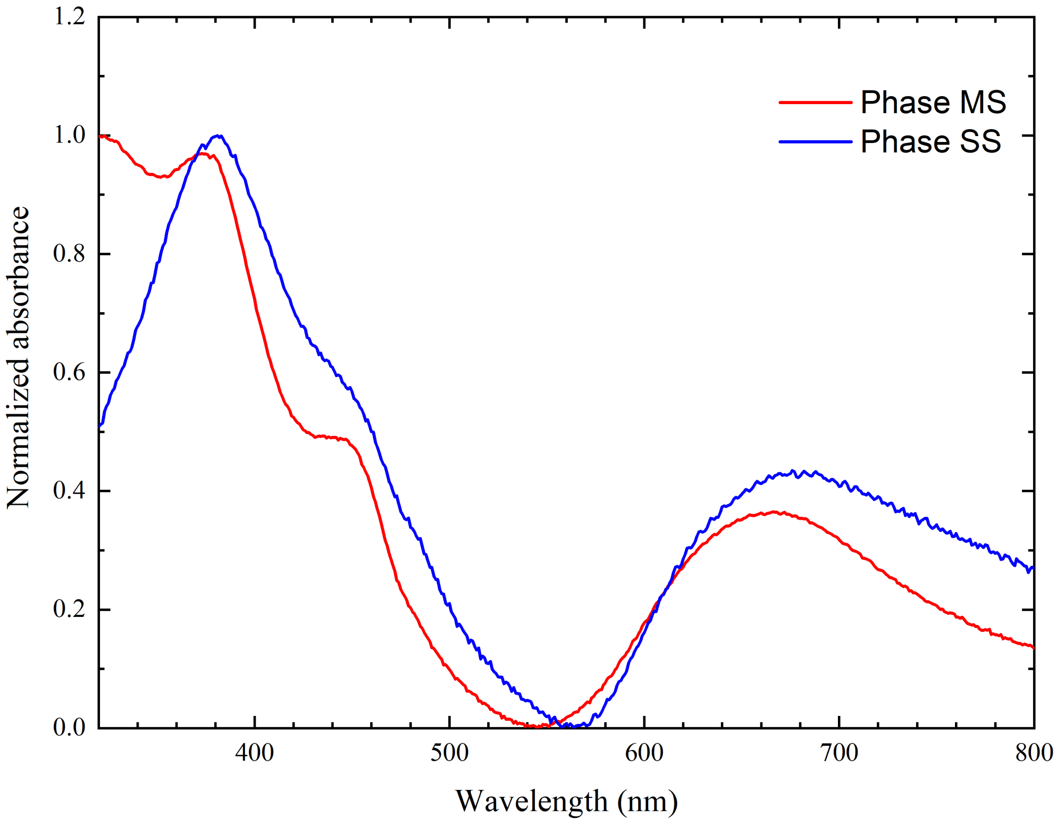

Figure S3: *Normalized UV-Vis spectra of the two N-TMB-TCNQF$_4$ polymorphs, ground on a quartz slide.*

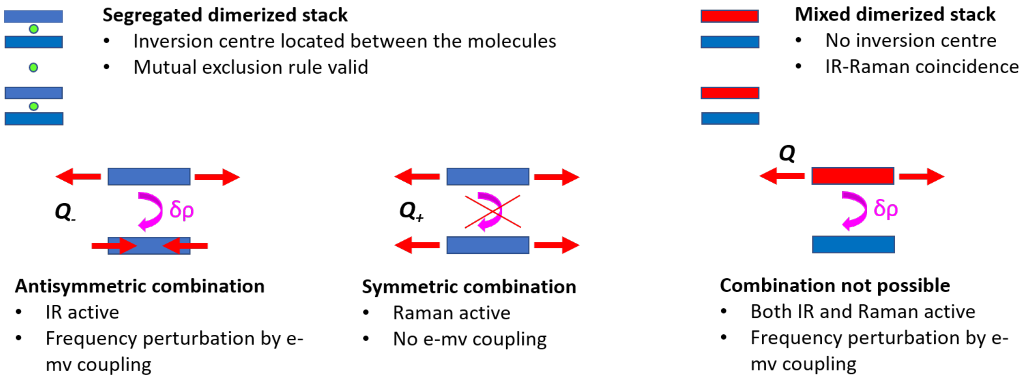

Figure S4: *e-mv coupling perturbation of the totally symmetric intramolecular vibrations in segregated and mixed dimerized stacks.*

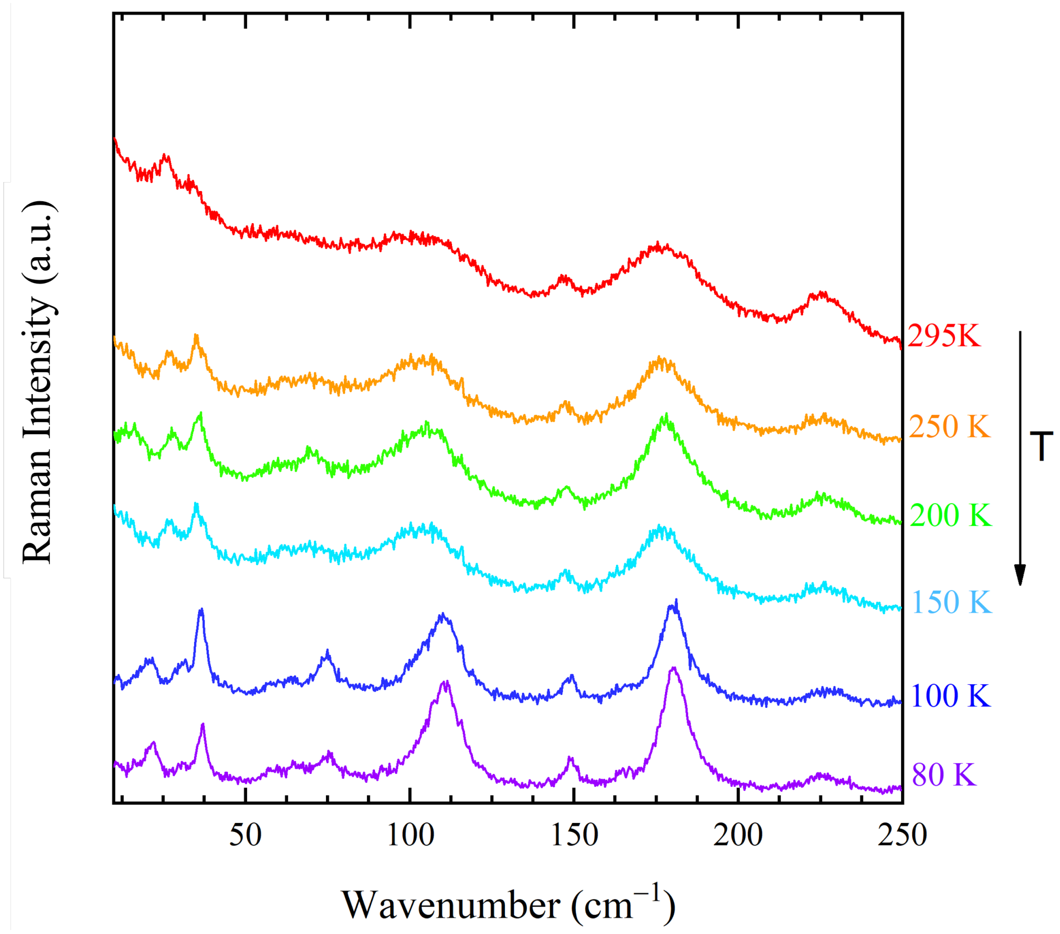

Figure S5: *Low temperature Raman spectra of N-TMB-TCNQF$_4$ Phase MS in the lattice phonon range. The exciting and scattered light are both polarized along z. The spectra are offset for clarity.*

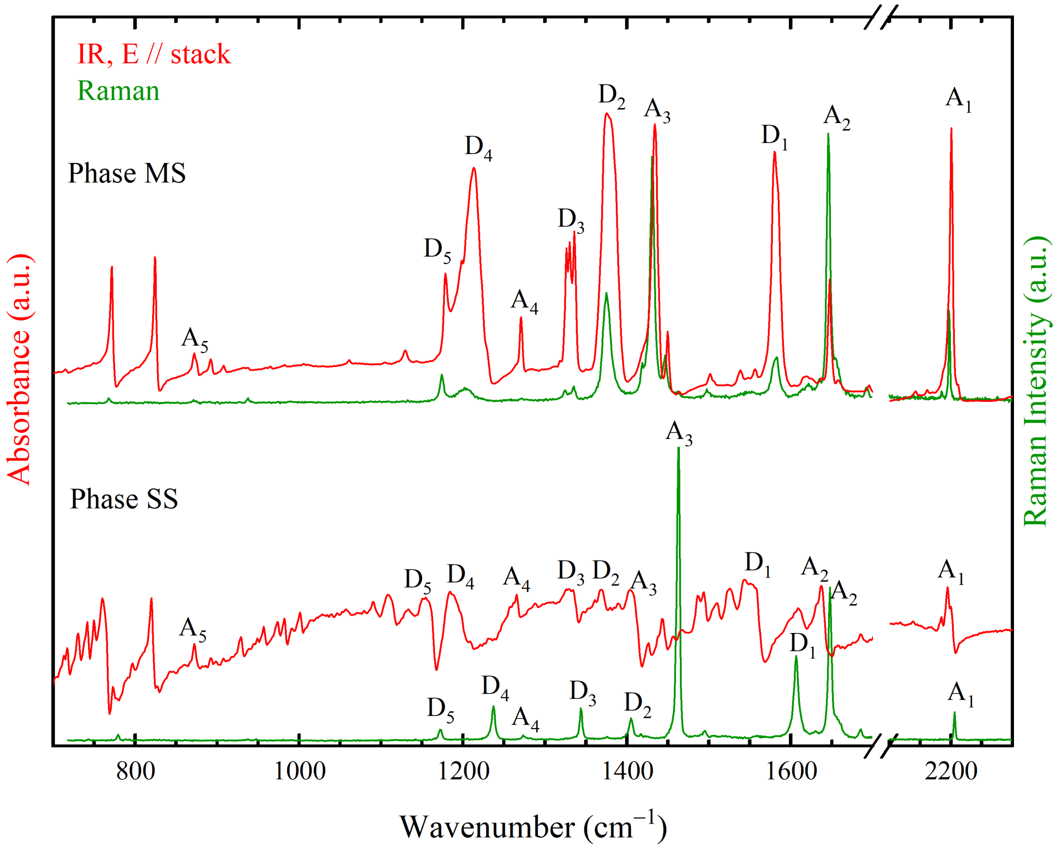

Figure S6: *Comparison between Raman and parallel polarized IR spectra of the two phases at 80 K. The letters correspond to the assignments reported in Table S1. D and A refer to Donor and Acceptor and indicate N-TMB and $TCNQF_4$ modes respectively. The two strong IR bands around 800 cm^{-1} are due to out-of-plane C-H and C-F bending modes of N-TMB and $TCNQF_4$. The intensities are scaled and the Phase MS spectra are upshifted for clarity.*

Table S1: *Frequencies of the totally symmetric modes in the Raman and parallel polarized IR spectra of the two polymorphs at 80 K. The assignments are based on Refs[1] and[2] for $TCNQF_4$ and N-TMB, respectively. All the frequencies are in cm^{-1} .*

Assignment<br>-	Phase MS<br>IR and Raman	Phase SS<br>IR	Phase SS<br>Raman
A_1: $TCNQF_4$, $a_g\nu_1$	2200	2194	2207
A_2: $TCNQF_4$, $a_g\nu_2$	1647	1638	1649
D_1: N-TMB, ν ring + δ CH (sym)	1580	1550	1607
A_3: $TCNQF_4$, $a_g\nu_3$	1434	1404	1464
D_2: N-TMB, ν N-ring (sym)	1377	1369	1405
D_3: N-TMB, ν ring-ring	1336	1331	1345
A_4: $TCNQF_4$, $a_g\nu_4$	1271	1266	1274
D_4: N-TMB, δ CH (sym)	1213	1187	1238
D_5: N-TMB, ρ CH_3	1178	1154	1173
A_5: $TCNQF_4$, $a_g\nu_5$	872	873	

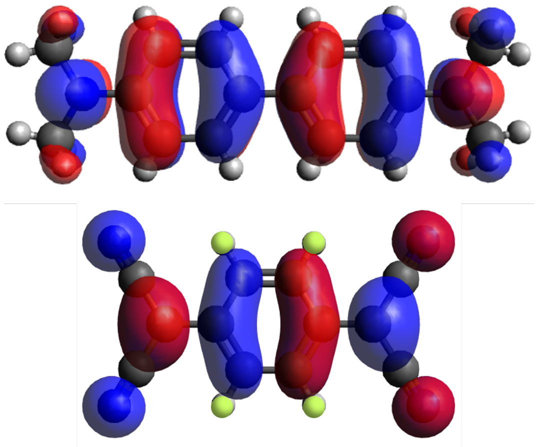

Figure S7: *N-TMB HOMO and $TCNQF_4$ LUMO, calculated by DFT, B3LYP/6-31G(d).*

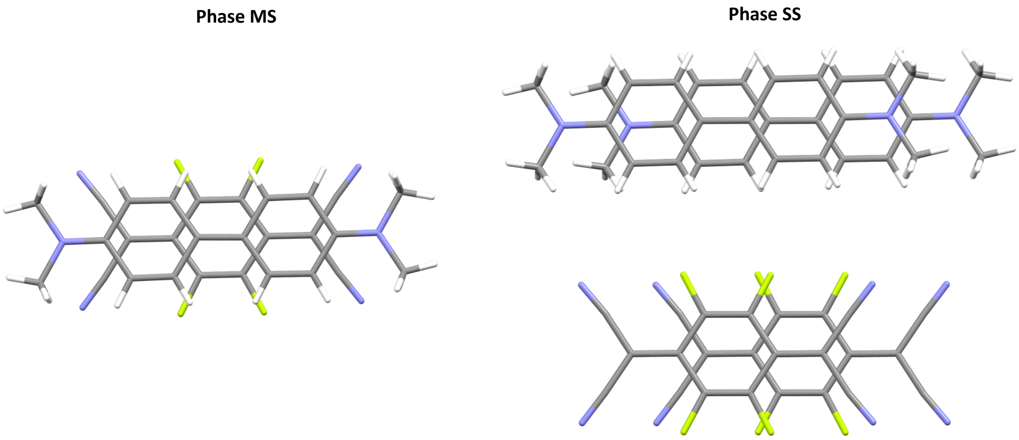

Figure S8: *Molecular overlap in the two phases, viewed perpendicular to the molecular plane. Left: D-A in Phase MS (the inter- and intra-dimer overlap appear the same as the molecular planes are perpendicular to the stack). Right: D-D and A-A in Phase SS.*

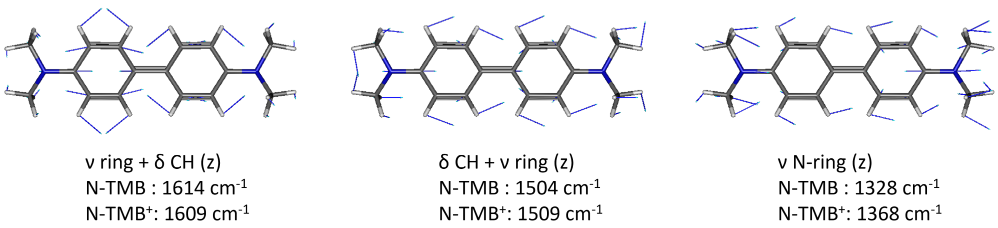

Figure S9: *Atomic displacements of the three strongest N-TMB IR bands, calculated by DFT, B3LYP/6-31G(d). These vibrations are long axis polarized and have a similar description in both the neutral molecule and its radical cation. The frequencies were scaled by the factor 0.9613, as suggested in.*[3] *Only the N-ring asymmetric stretching mode displays a large ionization frequency shift. The same occurs for the Raman active symmetric counterpart.*[2]

References

(1) Meneghetti, M.; Pecile, C. Charge–transfer organic crystals: Molecular vibrations and spectroscopic effects of electron–molecular vibration coupling of the strong electron acceptor TCNQF4. *The Journal of chemical physics* **1986**, *84*, 4149–4162.

(2) Boilet, L.; Buntinx, G.; Lapouge, C.; Lefumeux, C.; Poizat, O. Vibrational and structural analysis of the radical cation of N,N,N',N'-tetramethylbenzidine based on ab initio calculations and time-resolved resonance Raman spectroscopy. *Phys. Chem. Chem. Phys.* **2003**, *5*, 834–842.

(3) Merrick, J. P.; Moran, D.; Radom, L. An Evaluation of Harmonic Vibrational Frequency Scale Factors. *The Journal of Physical Chemistry A* **2007**, *111*, 11683–11700.
